# Phenotypic differentiation despite gene flow: Beak morphology, bite performance, and population genetics of Loggerhead Shrikes (*Lanius ludovicianus*)

**DOI:** 10.1002/ece3.11079

**Published:** 2024-03-19

**Authors:** Diego Sustaita, Gwendalyn K. Wulf, Arun Sethuraman

**Affiliations:** ^1^ Department of Biological Sciences California State University San Marcos San Marcos California USA; ^2^ Department of Biology San Diego State University San Diego California USA; ^3^ Present address: Beckman Center for Conservation Research San Diego Zoo Wildlife Alliance Escondido California USA

**Keywords:** beak morphology, bite performance, isolation by distance, Loggerhead Shrike, microsatellites

## Abstract

Previous studies of Loggerhead Shrikes (Laniidae: *Lanius ludovicianus*) in North America have indicated considerable intraspecific genetic and phenotypic differentiation, but the congruence between genetic and phenotypic differentiation remains obscure. We examined phenotypic differences in beak shape and bite force among geographic groupings across a 950 km range, from the lower Imperial Valley to the upper Central Valley of California, USA. We integrated these analyses with a population genetic analysis of six microsatellite markers to test for correspondence between phenotypic and genetic differences among geographic groups. We found significant phenotypic differentiation despite a lack of significant genetic differentiation among groups. Pairwise beak shape and bite force distances nevertheless were correlated with genetic (*F*_ST_) distances among geographic groups. Furthermore, the phenotypic and genetic distance matrices were correlated with pairwise geographic distances. Takentogether, these results suggest that phenotypic differences might be influenced by neutral processes, inbreeding (as indicated by high heterozygosity deficiencies we observed), local adaptation, and/or phenotypic plasticity.

## Graphical abstract

We examined differences in beak shape and bite force among geographic groupings of Loggerhead Shrikes across a 950 km range in California, USA. We integrated these data with an analysis of microsatellite markers to test for correspondence between phenotypic and genetic differences among geographic groups. We found significant phenotypic differentiation despite a lack of neutral genetic differentiation. Pairwise beak shape and bite force distances were nevertheless correlated with genetic distances among geographic groups, which in turn were correlated with pairwise geographic distances.

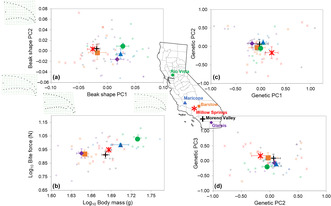

## 1 INTRODUCTION

Loggerhead Shrikes (*Lanius ludovicianus*) occupy a wide range of habitats, across which climate (e.g., temperature and precipitation) and prey resources (e.g., relative proportions of insects and vertebrates) vary considerably (Chabot & Lougheed, 2021; Craig, 1978; Morrison, 1980; Tyler, 1991; Yosef, 1996). Across their expansive North American distribution, Loggerhead Shrikes have historically been subdivided into 11 subspecies, based on morphology (mostly plumage coloration) and geography (Cade, 2011; Lefranc & Worfolk, 1997; Miller, 1931). Of these, seven are currently recognized (Yosef, 2008), many of which show some degree of genetic differentiation, particularly between eastern and western subspecies (Chabot, 2011; Mundy et al., 1997; Vallianatos et al., 2001, 2002).

Within California alone, multiple subspecies have been described based on both morphological (e.g., plumage coloration and bill and body dimensions; Cade, 2011; Miller, 1931; Patten & Campbell, 2000) and molecular (e.g., mitochondrial markers and nuclear microsatellites; Chabot, 2011; Chabot & Lougheed, 2021; Eggert et al., 2004; Mundy et al., 1997) differences, particularly between mainland and insular forms. Mainland subspecies in California traditionally included *L. l. gambeli*, *L. l. grinelli*, and *L. l. excubitorides* (encompassing Miller's [1931] *L. l. sonoriensis* and *L. l. nevadensis*; Cade 2011). However, Chabot and Lougheed's (2021) more recent comprehensive molecular analyses suggested no significant differentiation between *L. l. gambeli* and *L. l. mexicanus*, which was previously described as a resident of central and southern Mexico (Cade, 2011), and proposed that the two subspecies be considered a single subspecies under the name “*mexicanus*,” covering an expansive range from northwestern United States, through southern Mexico. As a result, subspecies, such as *L. l. mexicanus* (sensu Chabot & Lougheed, 2021), that enjoy a broad distribution throughout California (and beyond) are likely to show more localized patterns of phenotypic differentiation that mirror large‐scale subspecific differences. In fact, Sustaita and Rubega (2014) reported variation in upper bill shape and bite force – both of which ought to be important parameters of predatory proficiency – among Loggerhead Shrikes sampled throughout mid‐ and southern California. However, it is unclear whether these phenotypic differences are consistent with population‐level genetic differences found among subspecies, which may accrue over large geographic distances.

Here we uniquely integrate feeding‐related morphological and performance data with genetic data from the same individuals, to address whether variation in key traits for survival is attributable to population‐ or subpopulation‐level genetic differences. There are many different mechanisms by which genotypic and phenotypic differentiation can arise. We focused on the mechanisms of neutral genetic variation as a basis for establishing genetic differences among individuals from different locations, as well as to examine deviations in both genetic and phenotypic patterns from neutral expectations. Putatively neutral markers (e.g., Short Tandem Repeats or STRs such as microsatellites, minisatellites) are not expected to directly affect fitness traits, and are used extensively to study genetic diversity, differentiation (often measured using the *F*_ST_ statistic [Holderegger et al., 2006]), gene flow, and migration between populations. Thus, neutral markers provide a data set for assessing genetic similarities among individuals that is independent of the focal phenotypic data set. Furthermore, whereas neutral genetic divergence is typically attributed to gene flow and genetic drift, phenotypic divergence is often ascribed to natural selection (Chabot & Lougheed, 2021). Although selection can affect neutral DNA, and neutral processes like genetic drift, in turn, can affect phenotypes, genetic drift is typically taken as the null hypothesis for population differentiation (Spurgin et al., 2014).

We tested for phenotypic (beak shape and bite force) and genetic (using six microsatellite loci) differences among groups of Loggerhead Shrikes sampled from six different locations throughout California, encompassing a range of approximately 953 km. Our objective was to determine whether any phenotypic similarities and differences among geographic groupings are explained by genetic relationships, by leveraging the data set of Sustaita and Rubega (2014) on beak shape and bite force, combined with new analyses of linear morphometrics and population genetics. We hypothesized that, given the phenotypic variation previously observed among individuals across this geographic range (Sustaita & Rubega, 2014), there would be concomitant genetic differentiation in some neutral markers that are ostensibly independent of functional traits. Secondly, we tested for correlations among phenotypic, genotypic, and geographic distances among groupings, to test for patterns of isolation by distance. By exploring patterns of phenotypic and genetic differentiation among shrikes across various localities, we begin to isolate the potential mechanisms for geographic variation in feeding‐related phenotypic traits observed among geographic groups.

## 2 METHODS

### 2.1 Study area and field procedures

**FIGURE 1:**
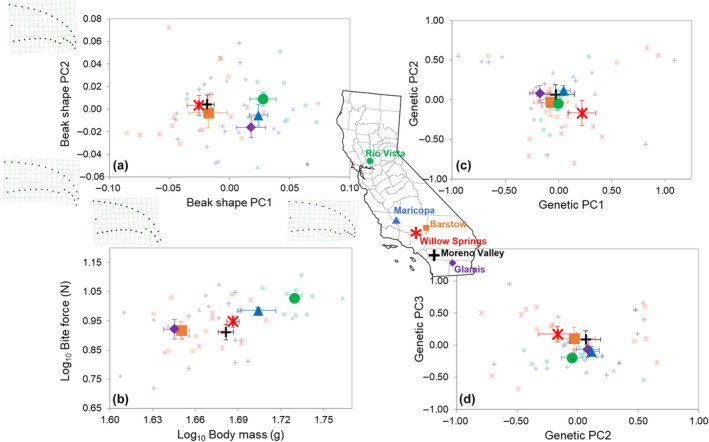
Location means ± SE along bivariate phenotypic (left side) and genetic (right side) spaces: (a) The first two principal component axes of upper beak shape, including shape deformation grids showing shape differences along the extremes of each axis; (b) log_10_ bite force vs. log_10_ body mass; (c) the first two axes of the genetic principal component analysis based on microsatellite data; (d) the second and third axes of the genetic PCA. Symbol color and shape combinations correspond to different sampling locations throughout California labeled on the map (individual data points are semi‐transparent). *Map source*: https://commons.wikimedia.org.

The study area and shrike trapping, handling, and measurement methods are detailed in Sustaita and Rubega (2014) and Sustaita et al. (2014). Briefly, we conducted field work in six general locations in California, USA, encompassing a linear distance of approximately 950 km (Figure 1): Willow Springs/Rosamond (approximately N 34°52.163′, W 118°30.346′); Maricopa (N 35°7.9112′, W 119°25.436′); Barstow/Hinkley (N 34°59.360′, W 117°17.787′); Glamis/Niland (N 33°10.765′, W 115°23.119′); Rio Vista (N 38°11.832′, W 121°46.589′); and Moreno (N 33°52.291′, W117°7.029′). These locations were chosen without any particular purpose or bias, other than for their relatively high shrike abundances, accessibility, and general differences in elevation, topography, and habitat. These locations are characterized by a Mediterranean climate, with a mean (±SD, based on *n* = 18 trap sites within and among locations) monthly high temperature of 25.2 ± 2.8°C, a mean monthly low of 10.1 ± 1.6°C, and a mean monthly precipitation of 20.3 ± 9.7 mm (based on 30‐year monthly averages from the National Climatic Data Center [http://www.ncdc.noaa.gov], via the Weather Channel [http://www.weather.com], averaged over 12 months and across trap sites). Shrikes were captured within the ranges of one to three putative subspecies, depending on the reference (*Lanius ludovicianus gambeli*, *L. l. sonoriensis*, and *L. l. nevadensis* of Miller [1931]; *L. l. gambeli* and *L. l. excubitorides* of Yosef [1996]; or *L. l. mexicanus* of Chabot & Lougheed [2021]). These subspecies, and more specifically the shrikes that inhabit the latitudinal range of our study, are primarily resident (Yosef, 1996, 2008). However, we cannot exclude the possibility that some winter migrants were intermixed with the resident birds that we captured (e.g., Pérez & Hobson, 2007; Chabot et al., 2018).

We then took a series of morphological and bite performance measurements, both sets of which were obtained for *n* = 57 birds, described and presented in greater detail by Sustaita and Rubega (2014). Briefly, we induced each shrike to bite modified force and pressure transducers (three times each) to measure jaw strength, both in terms of absolute force (N), and force per unit bill contact area (N/cm^2^), respectively (Sustaita & Rubega, 2014). We recorded sex and age based on plumage characteristics (following Pyle, 1997), then later confirmed sex genetically from breast feathers (Sustaita et al., 2014). We took a series of linear caliper measurements (to ±0.01 mm; Absolute Digimatic, Mitutoyo, USA) following Patten and Campbell (2000), among which included in this study are: bill length (nalospi; from the rostral end of the naris to the rostral upper bill tip), bill width and depth at the nares, hook tip length, tomial notch aperture, tarsus length (from the caudal end of the intertarsal joint to foot pad, between toes 1 and 2), folded wing chord, and tail length (measured dorsally, from the base to the distal central rectrices) using a 10‐cm ruler. We then took lateral‐view photos (using an Olympus Stylus 850 SW 8.0 MP digital camera) of the head and bill for subsequent 2D geometric morphometric analysis of upper bill shape (Sustaita & Rubega, 2014). Finally, we gently plucked three breast feathers from each individual and placed in uniquely labeled Ziplock pill pouch baggies and kept on ice in the field and later stored at −20°C for downstream genetic analysis.

### 2.2 Geometric morphometric methods

We used geometric morphometrics (a multivariate statistical technique for quantifying, visualizing, and comparing complex patterns of shape variation among organisms; Zelditch et al., 2012) to quantify variation in upper bill shape, as detailed in Sustaita and Rubega (2014). Briefly, we used tpsDig2 (Rohlf, 2010a) to digitize six distinctive, and repeatably identifiable, features of the upper bill (the naris, rostral culmen hook tip, vertex of the tomial notch, and vertex of the tomial tooth), from the sagittal plane, scaled, digital images (figure 1b of Sustaita & Rubega, 2014). We then digitally traced the dorsal and ventral contours of the upper bill to generate an additional 24 sliding semi‐landmarks, spaced equidistantly amidst the six primary landmarks, where there were no fixed homologous structures to digitize (Adams et al., 2004). These landmark coordinates (Data S1) were then imported into tpsRelw (Rohlf, 2010b) to first perform a generalized Procrustes analysis to align the landmark configurations, by scaling, rotating, and translating them to isolate the effects of shape and compute the “consensus” configuration (i.e., the average landmark positions across Procrustes superimposed specimens) (Rohlf, 1993; Zelditch et al., 2012). During this process, the semi‐landmarks were slid along the curves circumscribing the culmen and tomium to optimally match the corresponding positions on the consensus configuration, without deviating from the outline of the curve (Adams et al., 2004), resulting in a new set of Procrustes coordinates. Finally, these are subjected to a principal component analysis (PCA) to generate shape variables, along which shape changes can be visualized in the form of deformation grids.

### 2.3 Genetic methods

We successfully extracted nuclear genomic DNA from *n* = 54 birds, which we initially used for genetically sexing individuals (Sustaita et al., 2014). We then performed additional extractions according to the procedure outlined in the NuceloSpin Tissue XS extraction kit (Macherey Nagel). DNA quality and quantity were assessed through agarose gel electrophoresis and on a Qubit fluorometer 2.0 using broad range standards. Six fluorescently labeled (6‐FAM, Dye set DS‐33) pairs of microsatellite primers (LLU11, LLU20, LLU40, LLU45, LLU112, and LLU176) (Coxon et al., 2012) were used in amplification via polymerase chain reaction (hereafter referred to as PCR) in two set cycles dependent on the loci. We acknowledge that the number of markers used in this study limits our ability to resolve population genetic differences, and pales in comparison to the 17 microsatellite loci characterized by Coxon et al. (2012) and the 15 used by Chabot and Lougheed (2021). Nevertheless, studies of other birds have successfully used fewer than 15 markers to examine genetic differentiation among populations (10 by Cohen & Dor, 2018; seven by Eggert et al., 2004; Graham et al., 2020), even at a geographic scale comparable to this study (Graham et al., 2020). Additionally, several empirical studies have highlighted the effectiveness of inferring population structure (Liu et al., 2005), genetic diversity (Queirós et al., 2015), and heterozygosity (Väli et al., 2008) using random microsatellite markers across the genome. The thermal cycler conditions for loci LLU11 through LLU45 were: denaturation for 30 s at 98°C; 30 cycles of 98°C for 10 s, primer annealing at 50.2°C for 30 s, and extension at 72°C for 30 s, followed by a final extension of 72°C for 10 min. The thermal cycler conditions for loci LLU112 and LLU176 were: denaturation for 30 s at 98°C; 30 cycles of 98°C for 10 s, primer annealing 49°C for 30 s, and extension at 72°C for 30 s, followed by a final extension of 72°C for 10 min. These PCR products were then genotyped (Data S2) using capillary electrophoresis at Retrogen Inc., San Diego, CA, using GS600LIZ size standards. Fragment analysis data that was received from Retrogen Inc. was analyzed and individual genotypes were called by two separate individuals and conflicting peaks resolved by a third impartial individual using Genieous v.2019.0 (Kearse et al., 2012). One locus (LLU15) was removed from the analysis based on its null allele frequency >0.2 (Chapuis & Estoup, 2007).

### 2.4 Statistical analyses

To test for differences in beak shape among the six geographic locations from which shrikes were sampled, we used Procrustes ANOVA, implemented in function *procD.lm* of R Package “geomorph” (Adams et al., 2020). This approach leverages “residual randomization permutation procedures” to generate empirical null distributions to determine statistical significance (Adams & Collyer, 2018, 2019; Collyer & Adams, 2020). The models included log_10_ centroid size (i.e., “the square root of the sum of the squared distances of a set of landmarks from their centroid” Rohlf, 2010b); ANOVAs were based on type III Sums of Squares and Cross‐products and were set to 9999 permutations of null model residuals. This procedure tests for the effects of independent variables on the full set of dependent (geometric morphometric beak shape) variables (i.e., the Procrustes coordinates; the landmark coordinates aligned by generalized Procrustes analyses; Data S3). Sex and age differences in morphology and performance were not the focus of this study and are described by Sustaita and Rubega (2014). Nevertheless, we included effects of sex and age in the statistical analyses to account for these sources of variance when assessing differences among geographic groups.

We also used function *lm.rrpp* to test for differences in log_10_‐transformed peak bite force and body mass across locations (Data S3), accounting for sex and age. Bite force is often size‐dependent (Corbin et al., 2015; Sustaita & Rubega, 2014). Nevertheless, these could each respond independently to different geographic localities, and by considering them together we account for their potential interdependence. Finally, we performed a complementary set of multivariate analyses on the set of (log_10_‐transformed) linear morphometric measurements described above. The data were normally distributed, but because they failed to meet the assumption of equality of variance–covariance matrices for parametric MANOVA, and for the sake of consistency with the geometric morphometric analyses described above, we used the *lm.rrpp* function of R package “RRPP” (Collyer & Adams, 2018, 2020), which employs the same residual randomization approach described above, to test for differences among geographic groups. We caution that, strictly speaking, these geographic groups may not be statistically independent, given the variation in proximity between locations and the fact that shrikes are capable of moving great distance. However, any spatial autocorrelation would diminish differences among groups, and therefore make the tests more conservative. We used the *pr.comp* function of “stats” package in R (R Core Team, 2021) to perform a PCA to visualize the dispersion among groups.

We performed standard population genetic analyses for deviations from Hardy–Weinberg equilibrium (using Fisher's Exact tests), pairwise *F*_ST_ tests, and Fisher's exact G tests of genotypic differentiation among the six geographic groupings using Genepop On the Web v.4.7.5 (https://genepop.curtin.edu.au/). Statistical significance was assessed after the Benjamini‐Hochberg correction for multiple testing. Normalized genetic distances between locations were calculated as *F*_ST_/1 − *F*_ST_ (after Spurgin et al., 2014), and pairwise distances were encoded into a genetic distance matrix. We used the *dudi* function implemented in the “ade4” package in R (Dray & Dufour, 2007) to perform a genetic PCA on the six microsatellite loci to examine groupings by geographic location. To test for genetic differences among geographic groupings along the full set of *n* − 1 = 53 genetic principal components (PCs), we performed a multivariate analysis of variance using function *lm.rrpp* of R package “RRPP” (Collyer & Adams, 2020). Much like the *procD.lm* function described above, procedure uses empirical probability distributions of linear model statistics generated by randomizing residuals and adding them to fitted models to produce random “pseudovalues” (Collyer & Adams, 2018). Over several permutations, model coefficients and sums‐of‐squares are estimated for full models, resulting in empirical sampling distributions of ANOVA statistics for tests of effects (Collyer & Adams, 2018). Accordingly, the procedure is robust to high‐dimensional data sets, characterized by high *p* (number of variables): *n* (number of observations) ratios (Collyer & Adams, 2018).

Since all analyses that included genetic data were based on a relatively small number of microsatellite loci, we (1) assessed the power of our dataset to accurately estimate genetic diversity (here measured as heterozygosity) and inbreeding coefficients (*F*_IS_) within sampled locales as the squared correlation coefficient (*r* ^2^) between estimates of heterozygosity and *F*_IS_ across 1000 bootstrap replicates of our microsatellite data – we expected that a high congruence of the true *r* ^2^ and the simulated *r* ^2^ distribution would indicate statistical power to discern heterozygosity and inbreeding coefficients, and (2) assessed the sensitivity of the estimates of the squared correlation coefficient (*r* ^2^) between estimates of heterozygosity and *F*_IS_ with varying number of microsatellite loci from 2 to 10, across 1000 replicate simulations for each number of loci – we expected that if the simulated mean and confidence intervals included the “true” *r* ^2^, then there is sufficient statistical power to discern heterozygosity and *F*_IS_ using varying number of loci. All simulations and sensitivity analyses were performed using the “inbreedR” package in R (Stoffel et al., 2016).

To test for congruence between phenotypic and genetic data sets, we constructed matrices to compute pairwise phenotypic and genetic distances among geographic groupings (Data S3). We ran a PCA on the six upper bill landmarks and 24 sliding semi‐landmarks from digital images of the upper beak photographed in lateral view, following GPA superimposition (Sustaita & Rubega, 2014) using tpsRelwarp32 (Rohlf, 2010b), from which pairwise Procrustes distances among geographic groupings were computed using tpsSplin (Rohlf, 2021) to create a “morphological” distance matrix. The peak bite forces (N) and body masses (g) were averaged over shrikes from each geographic grouping. We then constructed a Bray‐Curtis distance matrix from the bivariate bite force and body mass data set (because they are interrelated) to generate a single, integrated “performance” distance matrix using R package “ecodist” (Goslee & Urban, 2007). For the “genetic” matrix, we used the pairwise genetic distances (*F*_ST_/1 − *F*_ST_) among shrikes sampled from each locality, described earlier. We then performed Mantel tests for correlations between matrices, and for patterns of isolation by distance (e.g., Spurgin et al., 2014) using the “ecodist” package in R (Goslee & Urban, 2007). Finally, we tested for correlations between location‐level inbreeding coefficients and location‐mean phenotypic variables using Spearman rank correlations using the *cor.test* function of base R (R Core Team, 2021).

## 3 RESULTS

The first three geometric morphometric beak shape components accounted for 82.4% of the variance among individuals across locations; PC1 explained 45.1% of the variance, and PC2 explained another 26.3% (Figure 1). PC1 primarily reflected variation in the relative length of the rostral hooked tip, increasing from negative to positive, whereas PC2 reflected variation in the relative length of the culmen, from long and gracile (negative) to short and stout (positive). After removing the non‐significant location × log_10_ centroid size interaction (Table 1), there were significant differences among locations (Procrustes ANOVA; *F*_5,49_ = 2.030, *p* = .0108; Table 1), accounting for significant variation in centroid size (*F*_1,49_ = 5.078, *p* = .0021; Table 1). Much of the differentiation among locations occurred between a cluster formed by Barstow, Moreno, and Willow Springs, vs. Rio Vista, Maricopa, and Glamis along PC1 (Figure 1).

**TABLE 1:** Results of multivariate *procD.lm* ANOVA testing for differences along the full set of upper beak shape morphometric variables while accounting for bill size (i.e., centroid size), sex, and age based on type III Sums of Squares and Cross‐products and randomization of null model residuals set to 9999 iterations.

	df	SS	MS	*R*_sq_	*F*	*Z*	Pr(>*F*)
Full model^a^							
Location	5	0.005752	0.00115043	.05885	0.7806	−0.61112	.7303
Log(Csize)	1	0.001600	0.00159988	.01637	1.0856	0.44361	.3322
Sex	1	0.002002	0.00200233	.02049	1.3586	0.75679	.2338
Age	1	0.000711	0.00071094	.00727	0.4824	−0.77735	.7744
Location:Log(Csize)	5	0.005786	0.00115711	.05920	0.7851	−0.59390	.7232
Residuals	44	0.064846	0.00147377	.66349			
Total	57	0.097735					
Reduced model							
Location	5	0.014631	0.0029262	.14970	2.0301	2.27302	.0108
Log(Csize)	1	0.007320	0.0073199	.07490	5.0781	2.72805	.0021
Sex	1	0.002350	0.0023496	.02404	1.6300	1.02978	.1522
Age	1	0.000627	0.0006267	.00641	0.4348	−0.93983	.8147
Residuals	49	0.070631	0.0014415	.72268			
Total	57	0.097735					

^a^ Full model procD.lm(coords ~ location * log(Csize) + sex + age, iter = 9999, SS.type = “III”).

Peak bite force scaled positively with body mass among individuals, and there was significant (multivariate) differentiation in bite force and body mass among groups of individuals across locations (Figure 1; RRPP ANOVA; *F*_5,48_ = 4.294, *p* = .0013; Table 2). There was a much clearer geographic pattern to bite force and body mass, both of which tended to increase from south to north (Figure 1).

**TABLE 2:** Results of multivariate *lm.rrpp* model^a^ testing for differences among locations along the performance set of variables (log_10_ bite force and log_10_ body mass), accounting for sex and age differences, based on type III Sums of Squares and Cross‐products and randomization of null model residuals set to 9999 iterations.

	df	SS	MS	*R*_sq_	*F*	*Z*	Pr(>*F*)
Location	5	0.12766	0.0255315	.28027	4.2944	2.94876	.0013
Sex	1	0.01465	0.0146490	.03216	2.4639	1.24104	.1182
Age	1	0.00269	0.0026891	.00590	0.4523	−0.06816	.5266
Residuals	48	0.28538	0.0059454	.62655			
Total	55	0.45547					

^a^ Model lm.rrpp(cbind(logbite, logmass) ~ location + sex + age, iter = 9999, SS.type = “III”).

**FIGURE 2:**
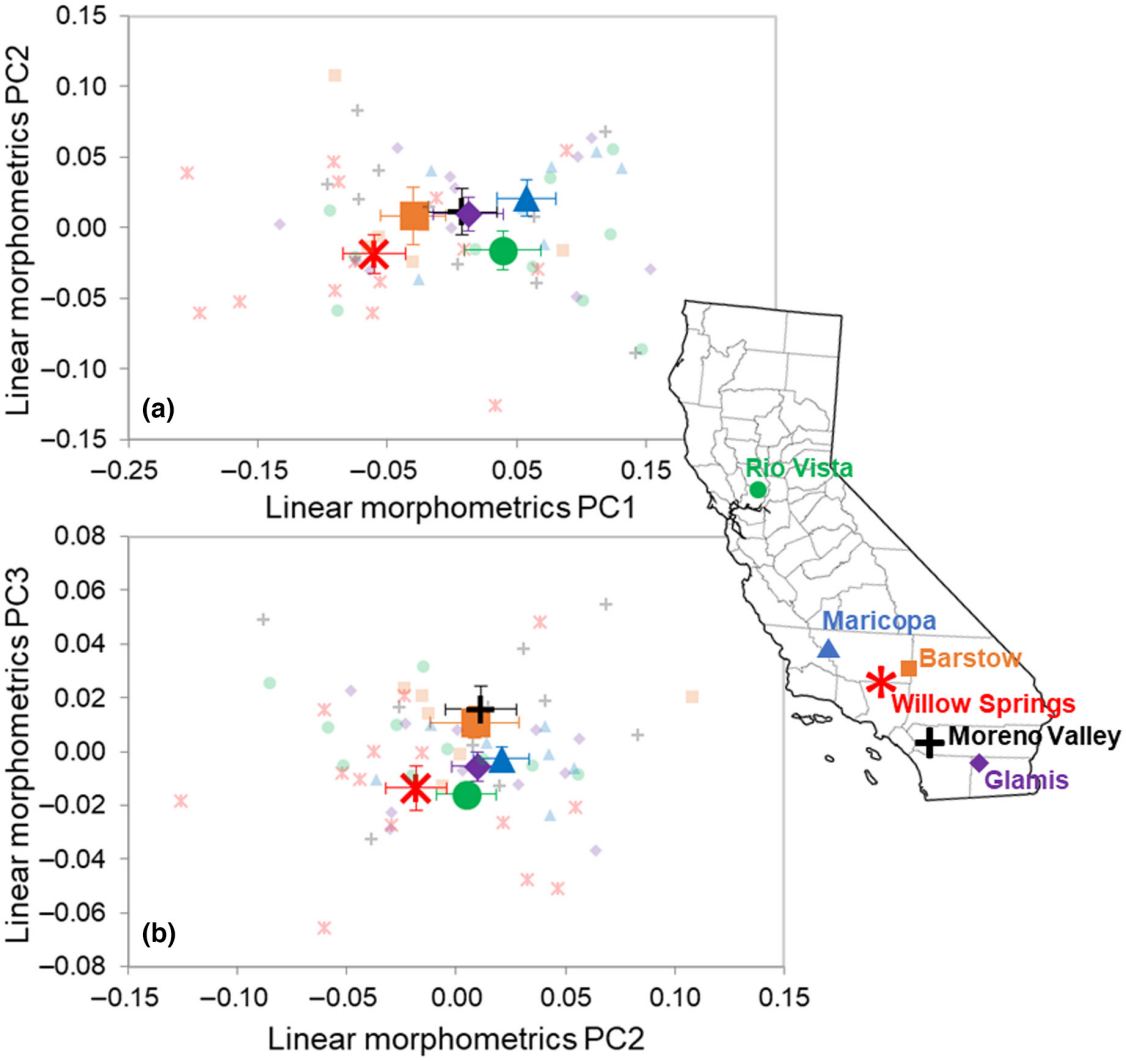
Location means ± SE along (a) the first two principal component axes from the full set of eight log_10_‐transformed linear morphometric variables, and (b) the second and third PC axes. Symbol color and shape combinations correspond to different sampling locations throughout California labeled on the map (individual data points are semi‐transparent). *Map source*: https://commons.wikimedia.org.

The more traditional linear morphometrics showed less differentiation among geographic groupings than did the bill shape variables (Figure 2), but nevertheless corroborated the morphological differences (*p* = .0173; Table 3; Figure 2). However, these differences were largely driven by the linear bill measurements. When the bill (nalospi, bill depth, bill width, hook length, and tomial tooth distance) and body (wing chord, tarsus length, and tail length) dimensions were analyzed separately, only the bill dimensions model was significant (*p* = .0180; Table 3) and explained a more substantial amount of variance (*R* ^2^ = .195) than did the body dimensions model (*R* ^2^ = .084) (Table 3). This result is echoed in the component loadings; bill measurements, particularly hook length (−0.82), tomial tooth distance (−0.83), and bill width (−0.76), loaded highly on PCs 1, 2, and 3 (respectively; accounting for 92.9% of the variance), whereas the body measurements, particularly wing chord (0.49), tail length (0.53), and tarsus length (−0.64), loaded more highly on PCs 4–6.

**TABLE 3:** Results of multivariate *lm.rrpp* ANOVA testing for differences among locations (accounting for sex and age differences) along the set of log_10_‐transformed linear morphometrics, based on type III Sums of Squares and Cross‐products and randomization of null model residuals set to 9999 iterations.

	df	SS	MS	*R*_sq_	*F*	*Z*	Pr(>*F*)
All variables combined^a^							
Location	5	0.12781	0.0255619	.19036	2.3975	2.09456	.0173
Sex	1	0.00845	0.0084477	.01258	0.7923	0.15634	.4358
Age	1	0.00563	0.0056266	.00838	0.5277	−0.25629	.5947
Residuals	50	0.53311	0.0106621	.79402			
Total	57	0.67140					
Body metrics only (wing chord, tarsus length, tail length)							
Location	5	0.0026078	0.0005216	.08431	1.1185	0.3957	.3475
Sex	1	0.0052148	0.0052148	.16859	11.1837	3.3948	.0002
Age	1	0.0003195	0.0003195	.01033	0.6853	−0.0451	.5154
Residuals	50	0.0233143	0.0004663	.75375			
Total	57	0.0309311					
Bill metrics only (nalospi, bill depth, bill width, hook length, tomial tooth distance)							
Location	5	0.12520	0.0250403	.19548	2.4559	2.09319	.0180
Sex	1	0.00323	0.0032329	.00505	0.3171	−0.68485	.7505
Age	1	0.00531	0.0053071	.00829	0.5205	−0.22158	.5831
Residuals	50	0.50979	0.0101958	.79596			
Total	57	0.64047					

^a^ Model lm.rrpp(cbind(log_10_ wing chord, log_10_ tarsus length, log_10_ tail length, log_10_ nalospi, log_10_ bill depth, log_10_ bill width, log_10_ hook length, log_10_ ttdistance) ~ location + sex + age, iter = 9999, SS.type = “III”).

Pairwise population differentiation, estimated as Weir and Cockerham's *F*_ST_ throughout the six California study areas, were relatively low (Table 4), and Fisher's exact G tests indicated no significant genetic differentiation among groups of individuals among locations (all corrected *p* ≥ .10). The greatest pairwise *F*_ST_ values were associated with the Rio Vista groups, the furthest, northernmost location in northern California (Figure 1). Allele frequencies significantly deviated from Hardy–Weinberg for some loci in four of the six geographic groups (Glamis: *χ* ^2^ = 39.8, df = 12, *p* < .0001; Willow Springs: *χ* ^2^ = 40.0, df = 12, *p* < .0001; Moreno Valley: *χ* ^2^ = 58.6, df = 12, *p* < .0001; Rio Vista: *χ* ^2^ = 28.8, df = 12, *p* = .004). The relatively high inbreeding coefficients (Table 5) mirrored the generally low *F*_ST_ values, and the estimated rate of inbreeding within groups was highest in Glamis and Moreno Valley (Table 5). The genetic PCA on the microsatellite loci captured 90% of the variance in the first 38 components, but the scree plot showed a considerable drop in eigenvalues after the third PC (collectively accounting for 14.8% of the variance), and consequently we used these to visualize groupings in Figure 1. There was considerable clustering among locations between PC1 and PC2 and PC2 and PC3 (Figure 1), with the exception of the Willow Springs group which was isolated most along PC1. However, there was no significant differentiation among groups along the 53 genetic PCs collectively (RRPP ANOVA; *F*_5,48_ = 1.063, *p* = .124; Table 6).

**TABLE 4:** Pairwise *F*_ST_ values based on six microsatellite loci, among populations of Loggerhead Shrikes across six study areas throughout California.

Location	Glamis	Maricopa	Willow Springs	Moreno Valley	Barstow
Maricopa	0.0159				
Willow Springs	0.0113	0.0176			
Moreno Valley	−0.004	−0.0065	−0.0047		
Barstow	0.0028	0.0171	−0.0042	−0.0093	
Rio Vista	0.0187	0.0165	0.0252	0.0242	0.0268

*Note*: None of the pairs of populations were determined to be statistically significantly different from each other using the Fisher's exact G test implemented in Genepop v.4.7.5 (Benjamini‐Hochberg corrected *p*‐value of >.1).

**TABLE 5:** Identity‐based genetic diversities within (1‐Qintra) and among (1‐Qinter) individuals (per locus) and inbreeding coefficient (*F*_IS_) values of individuals within each general California location.

Location	1‐Qintra	1‐Qinter	*F*_IS_
Glamis	0.7609	0.9651	0.2117
Maricopa	0.8438	0.9437	0.106
Willow Springs	0.7692	0.9445	0.1856
Moreno Valley	0.7414	0.9664	0.2328
Barstow	0.8065	0.9599	0.1599
Rio Vista	0.775	0.9514	0.1854

**TABLE 6:** Results of multivariate *lm.rrpp* model^a^ testing for differences among locations along the full set of 53 genetic principal components (based on microsatellite data), based on type III Sums of Squares and Cross‐products and randomization of null model residuals set to 9999 iterations.

	df	SS	MS	*R*_sq_	*F*	*Z*	Pr(>*F*)
Location	5	14.492	2.8985	.09969	1.063	1.1437	.1242
Residuals	48	130.876	2.7266	.90031			
Total	53	145.369					

^a^ Model lm.rrpp(cbind(pc1…pc53) ~ location, iter = 9999, SS.type = “III”).

**FIGURE 3:**
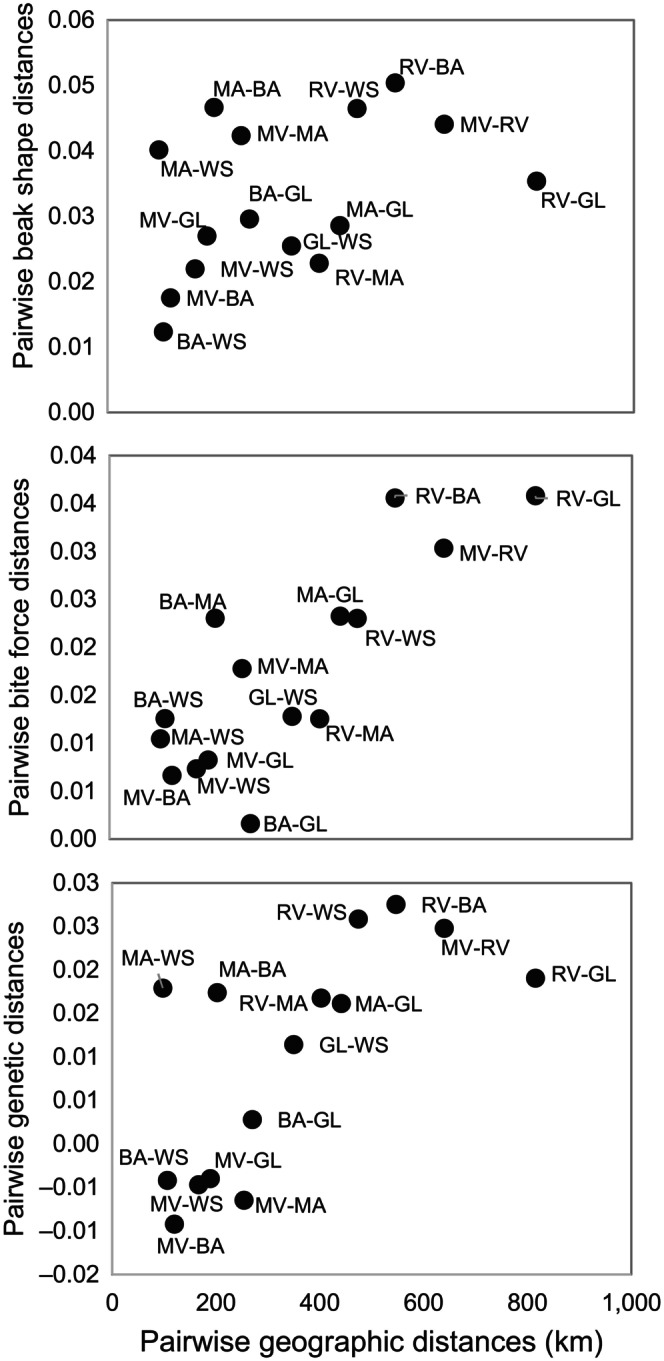
Pairwise morphological (top), performance (middle), and genetic (bottom) distances against pairwise geographic distances. Points represent pairwise differences in location means across six California locations sampled (BA, Barstow; GA, Glamis; MA, Maricopa; MV, Moreno Valley; RV, Rio Vista; WS, Willow Springs).

**FIGURE 4:**
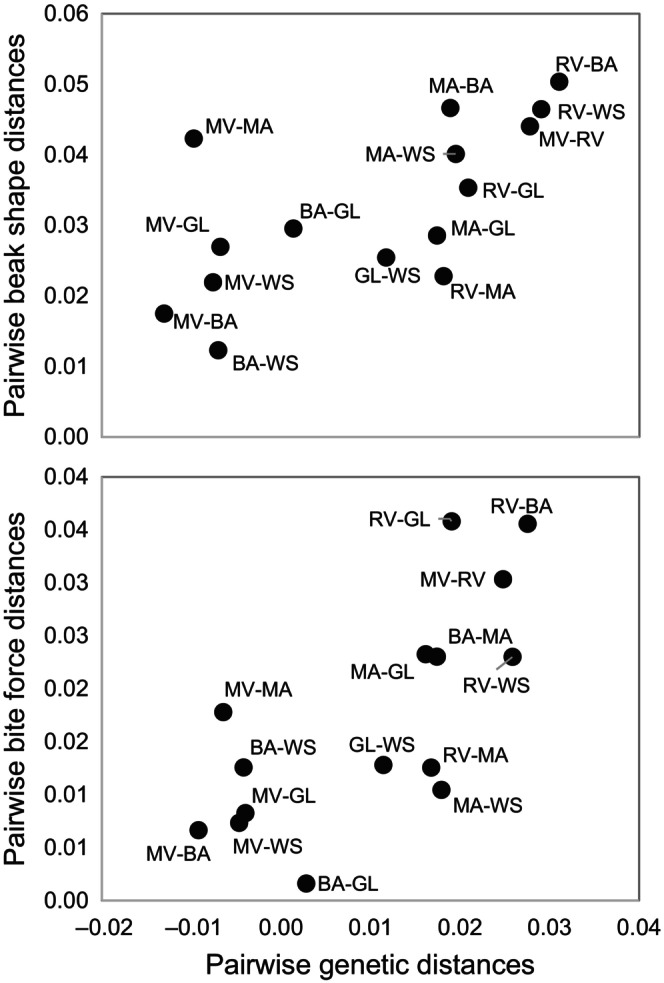
Pairwise morphological (top) and performance (bottom) distances against pairwise genetic distances. Points represent pairwise differences in location means across six California locations sampled (BA, Barstow; GA, Glamis; MA, Maricopa; MV, Moreno Valley; RV, Rio Vista; WS, Willow Springs).

Beak shape (Mantel *r* = .43, *p* = .037), bite force (Mantel *r* = .81, *p* = .004), and genetic distances (Mantel *r* = .688, *p* = .042) were each positively and significantly correlated with geographic distances (Figure 3). Similarly, beak shape (Mantel *r* = .68, *p* = .009) and bite force (Mantel *r* = .74, *p* = .013) distances were significantly, positively correlated with genetic distances (Figure 4). To test whether these latter patterns were driven by the underlying geographic gradient, we performed additional partial Mantel tests (Goslee & Urban, 2007) to address whether differences in beak shape and bite force among locations were related to genetic differences once the linear effects of geographic distances were removed. Mantel *r*_partial_ = .59, *p* = .019 for beak shape, and *r*_partial_ = .39, *p* = .171 for bite force, suggesting that bite performance differences, but perhaps not beak shape differences, may be driven by geographic distances. Location‐specific inbreeding coefficient (*F*_IS_) values were not significantly correlated with location‐mean phenotypic traits (*F*_IS_ vs. beak shape PC1: Spearman's *ρ* = 0.143, *n* = 6, *p* = .8028; *F*_IS_ vs. beak shape PC2: Spearman's *ρ* = −0.257, *n* = 6, *p* = .658; *F*_IS_ vs. peak bite force: Spearman's *ρ* = −0.543, *n* = 6, *p* = .297; *F*_IS_ vs. body mass: Spearman's *ρ* = −0.428, *n* = 6, *p* = .419).

**FIGURE A1:**
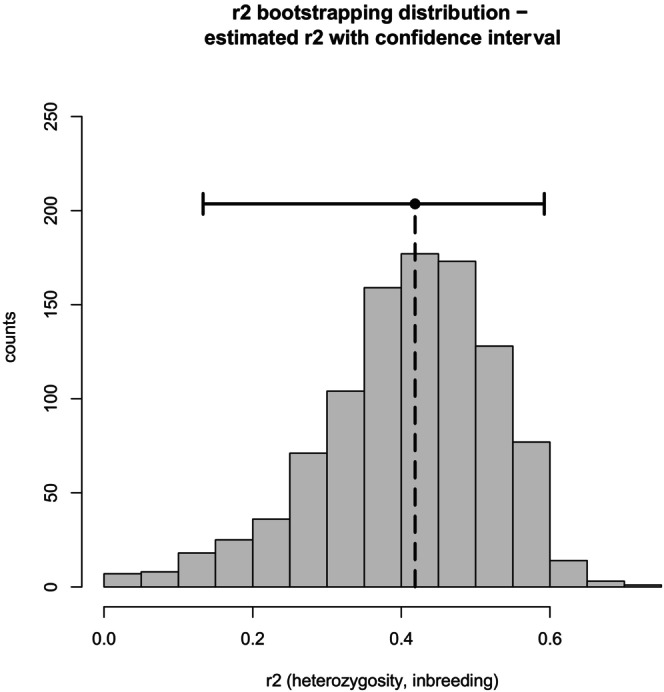
Distribution of correlations (*r* ^2^) between estimates of heterozygosity (genetic diversity) across 53 shrikes, at six microsatellite markers and estimates of their inbreeding coefficients (*F*_IS_) using 1000 bootstrap replicates, with the expected *r* ^2^ based on all markers estimated at 0.418.

**FIGURE A2:**
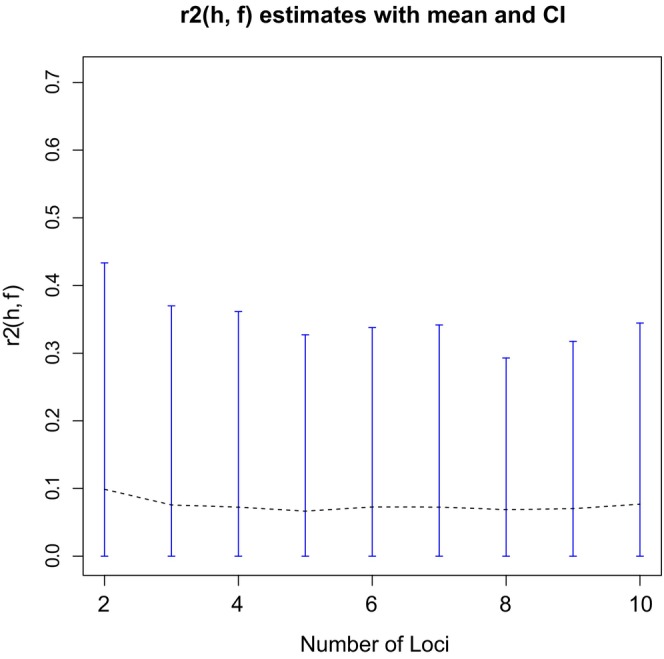
Sensitivity analyses for accurately estimating the correlation (*r* ^2^) between estimates of heterozygosity (genetic diversity) and inbreeding coefficients (*F*_IS_) by varying the number of sampled loci (2–10) but simulated in 53 individuals at a mean *F*_IS_ = 0.18 (var = 0.0016), as estimated from our six shrike geographic groups, across 1000 replicate simulations per scenario.

Power and sensitivity analyses of obtaining accurate estimates of heterozygosity and inbreeding coefficients (*F*_IS_) indicated (a) sufficient power within our dataset to discern low degree of population structure and high within‐population inbreeding (Appendix 1: Figure A1), and (b) sufficient sensitivity with six microsatellite markers to obtain accurate estimates of genetic diversity and inbreeding (Figure A2).

## 4 DISCUSSION

Our main goal was to determine whether any phenotypic similarities and differences among geographic groupings are explained by genetic relationships, and secondarily, to what extent phenotypic, genotypic, and geographic distances were correlated. Our results indicated no significant genetic differentiation among six general locations sampled haphazardly throughout California. This is not unexpected, given the relatively limited (~950 km) geographic range of the study, and in fact is consistent with studies of mainland Loggerhead Shrike subspecies conducted at larger scales (e.g., Chabot & Lougheed, 2021; Eggert et al., 2004). Nevertheless, we acknowledge that our results could be influenced by the small number of microsatellite loci we used. However, our power and sensitivity analyses of this dataset to inform extant levels of genetic diversity and inbreeding suggest the sufficiency of six microsatellite loci. Despite the lack of significant genetic differentiation, morphological and performance variation exists among groups from the localities sampled (Figures 1 and 2), with comparatively greater variation in bite force among localities.

The Mantel tests showed significant correlations between pairwise morphological and performance distances with genetic distances, suggesting that low degrees of population differentiation (albeit non‐significant) are enough to manifest potentially important phenotypic differences associated with feeding. These three distance matrices were also correlated with pairwise geographic distances, suggesting a model of isolation by distance, although this result is strongly influenced by the most distant geographic grouping of Rio Vista. Thus, groups that are further apart, and consequently less subject to gene flow, are more differentiated genetically and phenotypically. The distance matrix analyses provide some evidence for congruence between genetic and phenotypic differences. However, the PCA analyses show considerably more phenotypic, than genetic, variability among groups. Assuming that our low number of microsatellite loci sufficiently capture the variation among geographic groups, this result could indicate that the phenotypes might be influenced by a high rate of inbreeding (as suggested by high heterozygosity deficiencies), local adaptation, or phenotypic plasticity. We suggest that these plastic or adaptive responses and putative balancing selection in morphological and behavioral traits in response to local environmental conditions warrants further studies that involve genome‐wide analyses of phenotypic association.

All *F*_ST_ values were very low, indicating recent divergence and/or ongoing gene flow among individuals from different locations. Additionally, several of the loci analyzed indicated deviations from Hardy–Weinberg equilibrium. Taken together with the moderately high levels of inbreeding (and consequently high heterozygosity deficiencies) observed, this suggests that all six sampled locations comprise a single “subpopulation.” Chabot and Lougheed (2021) reported that genetic divergence was greatest between migratory and non‐migratory subspecies, and lowest among migratory populations geographically contiguous on a north–south axis. Although we intended to sample only resident populations according to Yosef (1996), we cannot exclude the possibility that fall and winter migrants from northern parts of the Loggerhead Shrike range were included among our samples, which naturally would facilitate gene flow (Chabot & Lougheed, 2021). However, to the extent that our inbreeding coefficients (*F*_IS_) reflect migratory (e.g., low values) vs. resident (e.g., high values) tendencies, there were no significant correlations between location‐specific *F*_IS_ values and location mean phenotypic values (beak shape PC1 and PC2; peak bite force, and body mass). Furthermore, there was no significant differentiation in wing, tail, and tarsus metrics that are often reflective of migratory tendencies (e.g., Chabot & Lougheed, 2021).

Previous work on shrikes has examined the congruence between morphological (standard linear morphometrics) and genetic (nuclear genetic and mtDNA markers) characteristics across subspecies on a much larger geographic scale than our study (Chabot & Lougheed, 2021). Chabot and Lougheed (2021) found that patterns of phenotypic variation generally mirrored those of genetic variation (i.e., low among migrants, high among residents), suggesting that selection for migration could lead to convergence of morphological traits (e.g., relatively shorter tails and longer wings) in migrants, that may override effects of local adaptation. Interestingly, the differences among geographic groupings in our much more localized study emanated primarily from bill, and not body, dimensions (e.g., Table 6). This suggests that perhaps at smaller geographic scales, ecological variation resulting from local adaptation is less likely to be obscured. Nevertheless, Chabot and Lougheed (2021) found greater genetic, than phenotypic, differences, and suggested that this pattern of incongruence may derive from rapid divergence in a few loci with key functional roles, while other parts of the genome experience greater admixture.

Conversely, other studies have uncovered phenotypic divergence in the face of low genetic differentiation in birds (as we have in our study), pointing to significant roles of selection and/or phenotypic plasticity (e.g., Cohen & Dor, 2018; Palacios et al., 2019; Phillimore et al., 2008). To this point, we estimated a heritability of beak shape (based on a parent‐offspring regression of bill shape axis PC1) in a captive population (*Lanius ludovicianus mearnsi*) of 0.57 (D. Sustaita, unpublished data), leaving ample room for environmental effects on phenotypes. Other studies have shown that drift, alone, is insufficient for explaining phenotypic divergences in spite of high gene flow (Clegg et al., 2002; Lima et al., 2012). Taken together with insights from these other studies, our results suggest that patterns of phenotypic variation among locations may be influenced by neutral processes (e.g., genetic drift), perhaps with some degree of selection and/or phenotypic plasticity operating on smaller scales within locations. Naturally the pattern of isolation by distance suggestive of neutral processes could be caused by population structure resulting from local adaptation. To this end, fine‐scale analyses of morphology‐performance relationships along an ecological gradient, as informed by carbon and nitrogen stable isotope analysis (Sustaita, 2013), are currently underway to shed more light on the importance of local adaptation (Sustaita et al., in prep.). Moreover, localized patterns of inbreeding, possibly owing to differences in migratory and dispersal patterns among geographic areas (e.g., Chabot & Lougheed, 2021), could also play a role in shaping genetic variation and local adaptation.

Both neutral processes and selection may both be important for variation among subspecies of shrikes (e.g., Chabot & Lougheed, 2021; Miller, 1931; Patten & Campbell, 2000), and perhaps among species (e.g., Gonzalez et al., 2008; Olsson et al., 2010; Peer et al., 2011; Sustaita, 2013). On the one hand, one might expect that phenotypic variation so closely aligned with feeding performance, such as beak shape and bite force, ought to be driven largely by selection and/or ecological plasticity, particularly in light of the plasticity of keratinous structures (Weiss & Kirchner, 2011). Naturally, more targeted genomic approaches, such as quantitative trait locus analysis or genome wide association mapping, are required to pinpoint the genes underlying these traits (e.g., Abzhanov et al., 2004, 2006; Knief et al., 2012) and to test for patterns of natural selection. On the other hand, neutral processes can directly affect genes underlying beak morphology and body size, resulting in differences in beak and body size of inbred individuals (Knief et al., 2012). Understanding the mechanisms of adaptation in the Loggerhead Shrike is important, given their rapid decline (among the top 10 most severe of North American land birds; Chabot & Lougheed, 2021). With one currently federally listed endangered subspecies (San Clemente Loggerhead Shrike), and other subspecies listed as threatened, endangered, or species of concern throughout several US states and Canada (Chabot & Lougheed, 2021; Pruitt, 2000), understanding their population structure (e.g., Caballero & Ashley, 2011; Rutledge et al., 2017) and how it affects their feeding‐related morphology and performance in the wild could have important implications for their conservation.

## AUTHOR CONTRIBUTIONS

**Diego Sustaita:** Conceptualization (lead); data curation (lead); formal analysis (lead); funding acquisition (lead); investigation (lead); methodology (lead); project administration (lead); resources (equal); software (equal); supervision (equal); validation (equal); visualization (lead); writing – original draft (lead); writing – review and editing (equal). **Gwendalyn K. Wulf:** Conceptualization (supporting); data curation (supporting); formal analysis (supporting); funding acquisition (supporting); investigation (supporting); methodology (equal); project administration (supporting); resources (supporting); software (supporting); supervision (supporting); validation (equal); visualization (equal); writing – original draft (equal); writing – review and editing (equal). **Arun Sethuraman:** Conceptualization (equal); data curation (equal); formal analysis (equal); funding acquisition (equal); investigation (equal); methodology (equal); project administration (equal); resources (equal); software (equal); supervision (equal); validation (equal); visualization (equal); writing – original draft (equal); writing – review and editing (equal).

## FUNDING INFORMATION

Financial and academic support for much of this work was provided by the CSU San Marcos Office of Graduate Studies and Research to DS, and the CSU San Marcos Summer Scholar's Program to GW.

## CONFLICT OF INTEREST STATEMENT

The authors declare no conflicts of or competing interests.

## Supporting information


Data S1.

Data S2.

Data S3.

## Data Availability

The data that support the findings of this study are available in the supporting information of this article.
